# An Extended FMEA Model Based on Cumulative Prospect Theory and Type-2 Intuitionistic Fuzzy VIKOR for the Railway Train Risk Prioritization

**DOI:** 10.3390/e22121418

**Published:** 2020-12-15

**Authors:** Yong Fu, Yong Qin, Weizhong Wang, Xinwang Liu, Limin Jia

**Affiliations:** 1 State Key Laboratory of Rail Traffic Control and Safety, Beijing Jiaotong University, Beijing 100044, China; 15120807@bjtu.edu.cn (Y.F.); lmjia@bjtu.edu.cn (L.J.); 2 School of Traffic and Transportation, Beijing Jiaotong University, Beijing 100044, China; 3 School of Economics and Management, Southeast University, Nanjing 211189, China; wangweizhongky@163.com (W.W.); xwliu@seu.edu.cn (X.L.)

**Keywords:** risk prioritization, type-2 IFNs, VIKOR, cumulative prospect theory, railway train operation

## Abstract

This paper aims toward the improvement of the limitations of traditional failure mode and effect analysis (FMEA) and examines the crucial failure modes and components for railway train operation. In order to overcome the drawbacks of current FMEA, this paper proposes a novel risk prioritization method based on cumulative prospect theory and type-2 intuitionistic fuzzy VIKOR approach. Type-2 intuitionistic VIKOR handles the combination of the risk factors with their entropy weight. Triangular fuzzy number intuitionistic fuzzy numbers (TFNIFNs) applied as type-2 intuitionistic fuzzy numbers (Type-2 IFNs) are adopted to depict the uncertainty in the risk analysis. Then, cumulative prospect theory is employed to deal with the FMEA team member’s risk sensitiveness and decision-making psychological behavior. Finally, a numerical example of the railway train bogie system is selected to illustrate the application and feasibility of the proposed extended FMEA model in this paper, and a comparison study is also performed to validate the practicability and effectiveness of the novel FMEA model. On this basis, this study can provide guidance for the risk prioritization of railway trains and indicate a direction for further research of risk management of rail traffic.

## 1. Introduction

As a kind of reliability analysis and risk management technique, failure mode and effect analysis (FMEA) has been widely used in rail traffic risk analysis [1,2]. In practice, the risk prioritization of every failure mode can be obtained by a risk priority number (RPN) through three risk factors of occurrence (O), severity (S) and detection (D). Then, the key components can be identified by the fusion of the RPN of each failure mode [3].

Despite the simplicity and understandability of the RPN method for the railway train risk analysis and prioritization, it still possesses many drawbacks. The most criticized drawbacks are presented as follows [4,5,6,7]:

- The multiplication for the RPN can be questionable and sensitive to the variations in risk factors calculation.
- Different combination of the risk factor O, S and D can produce the same RPN value, which is not effective in practical risk management.
- The risk factor O, S and D can be difficult to determined precisely in many real-world scenarios.
- The relative importance among the risk factor O, S and D can be overlooked in the conventional FMEA approach.

The most important and urgent business is to determine the criticality of the system component in the operation of railway train. Later, the maintenance optimization and improvement can be put into practice for the safety and reliability operation.

As stated above, there is a pressing need to further improve the conventional FMEA approach for the railway train risk analysis and prioritization. In order to overcome the shortcomings of traditional FMEA approach, some applications employed the risk information judgments with linguistic terms of fuzzy set theory. Triangular fuzzy numbers [8,9], interval fuzzy numbers [10], hesitant fuzzy numbers [11,12] and intuitionistic fuzzy numbers (IFNs) [13,14] have been adopted with MCDM methods to deal with the risk prioritization problems. By comparison, IFNs can be characterized by the membership function (MF) and non-membership function (NMF) to illustrate the positive and negative degree, respectively, which can be more flexible than other fuzzy terms. Nevertheless, it can be a challenge to determine the accurate data of their MFs and NMFs. Hence, type-2 IFNs have been put forward to handle the fuzzy problems in a way. Type-2 IFNs possess many advantages over IFNs, as their MFs and NMFs are themselves fuzzy, making it possible to model and minimize the effects of indeterminacy in fuzzy matters. Yu, Wang and Wang [15] extended IFNs with interval numbers as an application of type-2 IFNs for the uncertainty in the site-selection problem. Wei et al. [16] applied interval valued IFNs with entropy measures to overcome the existing entropy matters. However, the terminal point of MFs and NMFs can be difficult to determine and further enlarge the interval area after arithmetic operation. Therefore, Liu and Yuan [17] proposed TFNIFNs as another application of type-2 IFS to describe uncertainty and fuzziness. Compared with interval valued IFNs, TFNIFNs [18] can be better utilized in the fields of fuzziness as the triangular fuzzy number between 0 and 1, which can make TFNIFNs more flexible and reasonable.

On the other hand, some papers regarded the risk prioritization as a multi-criteria decision making (MCDM) problem [19,20,21,22,23,24]. Consequently, the MCDM model has been widely applied to solve the drawbacks in FMEA. Among the different MCDM technologies, TOSPIS and VIKOR methods are the widely used applications. Liu et al. [25] developed FMEA and VIKOR method to identify the risk of general anesthesia process. Lo et al. [26] proposed a novel FMEA model based on TOPSIS method for the equipment product risk identification. Furthermore, Mandal et al. [27] present a FMEA framework with fuzzy VIKOR approach for safety critical resources identification and risk mitigation purposes. Li et al. [28] tried to identify, evaluate and eliminate potential failures of the spindle box system by an advanced FMEA combined with fuzzy TOPSIS. According to the above researches, the solution obtained by the VIKOR method [29] is an aggregation of all the criteria, the relative importance of the criteria, and a balance between total and individual satisfaction. However, the solution determined by TOPSIS method considers the distances from the ideal point and negative-ideal point without considering their relative importance. Therefore, the comparison cases indicate that the VIKOR method can be slightly better than the TOPSIS method [30,31].

Although, to some extent, these efforts have eliminated the shortcomings of the conventional FMEA, there is a crucial issue that has not been fully coped with, namely, experts’ risk sensitiveness and decision-making psychological behavior. In order to solve this problem, prospect theory [32,33,34,35] combined with MCDM methods can be used to conduct risk prioritization in FMEA model by the consideration of experts’ risk sensitiveness and decision-making psychological behavior. However, traditional prospect theory also has the unacceptable drawback about violations of dominance. Consequently, cumulative prospect theory [36] has been proposed to handle this matter. In cumulative prospect theory, the probability weight is replaced by cumulative probability weight and makes it a clear logic as well as a relatively simple computation procedure, thus, cumulative prospect theory can be extensively applied in various MCDM problems [37,38,39,40].

According to the discussion above, in this paper, we develop an extended FMEA model based on cumulative prospect theory and type-2 intuitionistic fuzzy VIKOR for the railway train risk prioritization. In order to handle such situations where experts with different risk sensitiveness and decision-making psychological behavior towards different failure modes of railway train, cumulative prospect theory combined with TFNIFNs is adopted to depict the different risk sensitiveness and psychological behavior of experts. In addition, the VIKOR approach associated with entropy weight method is also carried out to fuse the risk information under different risk factors. Therefore, the final risk prioritization order can be obtained based on the compromise results of VIKOR. At last, a case study of railway train bogie system is utilized to illustrate the proposed extended FMEA model.

In the light of the above analysis, the contributions of this paper can be summarized as follows:

- The proposed risk component prioritization model based on FMEA framework considers all possible failure modes of railway train without losing any valid state information.
- The extended FMEA model combined with cumulative prospect theory considers the experts’ risk sensitiveness and decision-making psychological behavior which can obtain a relatively objective and reasonable risk prioritization outcome.
- The application of triangular fuzzy number intuitionistic fuzzy geometric (TFNIFG) operator as reference point can integrate all the risk score information comprehensively to determine the cumulative prospect value of each failure mode.

The rest of this paper can be organized as follows. In Section 2, the basic theories related to type-2 intuitionistic fuzzy numbers, VIKOR and cumulative prospect theory are briefly introduced. Section 3 presents the extended FMEA model based on cumulative prospect theory and type-2 intuitionistic fuzzy VIKOR for the railway train risk prioritization. In Section 4, a case study of the railway train bogie system is selected to illustrate the application and effectiveness of the proposed method. In Section 5, the conclusions and future research directions of this study are provided.

## 2. Preliminaries

This paper develops an extended FMEA model based on cumulative prospect theory and type-2 intuitionistic fuzzy VIKOR for the railway train risk prioritization. In this section, the basic concepts of TFNIFNs, VIKOR method and cumulative prospect theory are briefly introduced, which will be utilized in the subsequent sections.

### 2.1. TFNIFNs

**Definition** **1** ([41])**.** *Let X be a fixed set, an IFN A in X can be defined as*

(1) $$A=\left\{\langle x,u_{A}\left(x\right),v_{A}\left(x\right)\rangle |x\in X\right\}$$

*In which u_A_(x): X→[0, 1] and v_A_(x): X→[0, 1] is MF and NMF, respectively, satisfying 0 ≤ u_A_(x) + v_A_(x) ≤ 1, ∀x∈X. The number u_A_(x) and v_A_(x) shows the degree of MF and NMF of x to A, for all x∈X, respectively.*

**Definition** **2** ([18])**.** *A triangular fuzzy number C = (l, m, r) in X is a special fuzzy number, its MF can be defined as*

(2) $$u_{C}\left(x\right)=\left\{ \begin{array}{cc} \left(x-l\right)/\left(m-l\right), & l\leq x\leq m\\ \left(x-r\right)/\left(m-r\right), & m\leq x\leq r\\ 0, & otherwise \end{array}\right.$$

*In which 0≤l≤m≤r≤1, m is the barycenter of triangular fuzzy number C, if l = m = r, then C is an accurate number.*

*As for a triangular fuzzy number C, its fuzzy mean E(C) and standard deviation σ(C) can be defined as*

(3) $$E\left(C\right)=\frac{l+2m+r}{4}$$

(4) $$\sigma \left(C\right)=\frac{3l^{2}+4m^{2}+3r^{2}-4lm-2lr-4mr}{80}$$

**Definition** **3** ([18])**.** *Let $\tilde{A}=\left\{\langle x,u_{\tilde{A}}\left(x\right),v_{\tilde{A}}\left(x\right)\rangle |x\in X\right\}$ be a TFNIFN, and its MF $u_{\tilde{A}}\left(x\right)$ and NMF $v_{\tilde{A}}\left(x\right)$ is a triangular fuzzy number, respectively.*

*Let $\tilde{A}=\left\{\langle x,u_{\tilde{A}}\left(x\right),v_{\tilde{A}}\left(x\right)\rangle |x\in X\right\}$ be a TFNIFN, then the score function and variation function of $\tilde{A}$ can be defined as*

(5) $$S\left(\tilde{A}\right)=E\left(\mu_{\tilde{A}}\right)\frac{1+E\left(\mu_{\tilde{A}}\right)-E\left(\nu_{\tilde{A}}\right)}{2}\left(1-E\left(\mu_{\tilde{A}}\right)-E\left(\nu_{\tilde{A}}\right)\right)$$

(6) $$G\left(\tilde{A}\right)=\sigma \left(\mu_{\tilde{A}}\right)+\sigma \left(\nu_{\tilde{A}}\right)$$

*Let $\tilde{A_{1}}$ and $\tilde{A_{2}}$ be two TFNIFNs, we can define that:*

*If $S\left(\tilde{A_{1}}\right)<S\left(\tilde{A_{2}}\right)$, then $\tilde{A_{1}}<\tilde{A_{2}}$;*

*If $S\left(\tilde{A_{1}}\right)=S\left(\tilde{A_{2}}\right)\;and\;G\left(\tilde{A_{1}}\right)=G\left(\tilde{A_{2}}\right)$, then $\tilde{A_{1}}=\tilde{A_{2}}$;*

*If $S\left(\tilde{A_{1}}\right)=S\left(\tilde{A_{2}}\right)\;and\;G\left(\tilde{A_{1}}\right)>G\left(\tilde{A_{2}}\right)$, then $\tilde{A_{1}}<\tilde{A_{2}}$.*

**Definition** **4** ([18])**.** *Let ${\tilde{A}}_{i}=\left(\left(al_{i},am_{i},ar_{i}\right),\left(bl_{i},bm_{i},br_{i}\right)\right)$ be a set of TFNIFNs, i = 1, 2, …, N. The TFNIFG operator $\tilde{r}$ can be defined as*

(7) $$\begin{array}{l} \tilde{r}={\left({\tilde{A}}_{1}\right)}^{1/N}⊗{\left({\tilde{A}}_{2}\right)}^{1/N}⊗\cdots ⊗{\left({\tilde{A}}_{N}\right)}^{1/N}\\ =(\left(\prod_{i=1}^{N}{\left(al_{i}\right)}^{1/N},\prod_{i=1}^{N}{\left(am_{i}\right)}^{1/N},\prod_{i=1}^{N}{\left(ar_{i}\right)}^{1/N}\right),\\ \left(\left(1-\prod_{i=1}^{N}{\left(1-bl_{i}\right)}^{1/N}\right),\left(1-\prod_{i=1}^{N}{\left(1-bm_{i}\right)}^{1/N}\right),\left(1-\prod_{i=1}^{N}{\left(1-br_{i}\right)}^{1/N}\right)\right)) \end{array}$$

**Definition** **5** ([42])**.** *Let ${\tilde{A}}_{1}=\left(\left(al_{1},am_{1},ar_{1}\right),\left(bl_{1},bm_{1},br_{1}\right)\right)$ and ${\tilde{A}}_{2}=\left(\left(al_{2},am_{2},ar_{2}\right),\left(bl_{2},bm_{2},br_{2}\right)\right)$ be two TFNIFNs. The Hamming distance between ${\tilde{A}}_{1}$ and ${\tilde{A}}_{2}$ can be defined as*

(8) $$d\left({\tilde{A}}_{1},{\tilde{A}}_{2}\right)=\frac{1}{8}(\left|al_{1}-al_{2}\right|+2\left|am_{1}-am_{2}\right|+\left|ar_{1}-ar_{2}\right|+\left|bl_{1}-bl_{2}\right|+2\left|bm_{1}-bm_{2}\right|+\left|br_{1}-br_{2}\right|)$$

### 2.2. VIKOR Approach

The VIKOR approach proposed by Opricovic in 1998 is a widely used MCDM technique [27]. VIKOR approach is a compromise sorting method that can compromise ranking of a finite decision scheme by maximizing group utility and minimizing individual regret. The key idea of the VIKOR method is to select the optimal solution based on the closeness degree by each evaluation value, positive ideal solution and negative ideal solution. The procedure of VIKOR approach can be given below.

Step 1: Determine the positive ideal solution $f_{{}_{j}}^{+}$ and negative ideal solution $f_{{}_{j}}^{-}$ from the evaluation matrix.

(9) $$f_{{}_{j}}^{+}=\max f_{ij},f_{{}_{j}}^{-}=\min f_{ij}$$

Step 2: Determine the maximizing group utility *S_i_* and minimizing individual regret *R_i_* from the evaluation matrix.

(10) $$S_{i}=\sum_{j=1}^{m}w_{j}\frac{d\left(f_{{}_{j}}^{+},f_{ij}\right)}{d\left(f_{{}_{j}}^{+},f_{{}_{j}}^{-}\right)}$$

(11) $$R_{i}=\underset{j}{\max}\;w_{j}\frac{d\left(f_{{}_{j}}^{+},f_{ij}\right)}{d\left(f_{{}_{j}}^{+},f_{{}_{j}}^{-}\right)}$$

In which *w_j_* is the weight of criteria.

Step 3: Determine the compromise solution *Q_i_*.

(12) $$Q_{i}=\nu \frac{\left(S_{i}-S^{*}\right)}{\left(S^{-}-S^{*}\right)}+\left(1-\nu \right)\frac{\left(R_{i}-R^{*}\right)}{\left(R^{-}-R^{*}\right)}$$

In which *v* is the weight of the strategy of “the majority of criteria’’. The value of *v* lies in the range of 0–1. Normally, the value of *v* can be considered as 0.5. The intermediate parameters S^*^, *S*^−^, R^*^ and *R*^−^ can be given below.

(13) $$S^{*}=\underset{i}{\min}\;S_{i},\;S^{-}=\underset{i}{\max}\;S_{i},\;R^{*}=\underset{i}{\min}\;R_{i},\;R^{-}=\underset{i}{\max}\;R_{i}$$

Step 4: Determine the prioritization order of alternatives according to ascending order by the compromise solution *Q_i_*.

### 2.3. Cumulative Prospect Theory

Cumulative prospect theory put forward by Tversky and Kahneman in 1992 is an extended method of prospect theory [36]. It is a useful tool for assessment and decision making under risk and uncertainties in consideration of experts’ risk sensitiveness and decision-making psychological behavior. The significant enhancement of cumulative prospect theory compared with prospect theory is that the weight function is an inverse S-type curve. In cumulative prospect theory, the cumulative prospect value *V* can be determined as

(14) $$V=\sum_{i=-m}^{0}v\left(\Delta x_{ij}\right)\Pi_{i}^{-}+\sum_{i=1}^{n}v\left(\Delta x_{ij}\right)\Pi_{i}^{+}$$

In which $v\left(\Delta x_{ij}\right)$ is the value function and demonstrates the gains or losses of the alternative. It can be determined as:

(15) $$v\left(\Delta x_{ij}\right)=\left\{ \begin{array}{l} {\left(x_{ij}-x_{j}\right)}^{\alpha },x_{ij}\geq x_{j}\\ -\lambda {\left(x_{ij}-x_{j}\right)}^{\beta },x_{ij}<x_{j} \end{array}\right.$$

where *x_ij_* is the outcome of the alternative *ij*, *x_j_* is the reference point of the alternative *ij* under the criterion *j*. *α* and *β* are the exponential parameters related to gains and losses. *λ* is the risk aversion parameter, and it represents the characteristic of steeper for losses than for gains. Based on the experiment validation [36], these parameters can be provided as *α* = *β* = 0.88, *λ* = 2.25.

$\Pi_{i}^{-}$ and $\Pi_{i}^{+}$ are the decision weight and can be expressed as follows when the value function $v\left(\Delta x_{ij}\right)>0$ and $v\left(\Delta x_{ij}\right)<0$, respectively.

(16) $$\Pi_{i}^{+}=w^{+}\left(p_{i}+p_{i+1}+\cdots +p_{n}\right)-w^{+}\left(p_{i+1}+p_{i+2}+\cdots +p_{n}\right)$$

(17) $$\Pi_{i}^{-}=w^{-}\left(p_{-m}+p_{-m+1}+\cdots +p_{i}\right)-w^{-}\left(p_{-m}+p_{-m+1}+\cdots +p_{i-1}\right)$$

where *w*^+^ and *w*^−^ are the weight functions and are strictly increasing functions from the unit interval into itself. The weight function *w*^+^ and *w*^−^ can be expressed as

(18) $$w^{+}\left(p\right)=\frac{p^{\gamma }}{{\left[p^{\gamma }+{\left(1-p\right)}^{\gamma }\right]}^{\frac{1}{\gamma }}}$$

(19) $$w^{-}\left(p\right)=\frac{p^{\delta }}{{\left[p^{\delta }+{\left(1-p\right)}^{\delta }\right]}^{\frac{1}{\delta }}}$$

where *γ* and *δ* are the attitude parameters for gains and losses. Generally, the values of *γ* and *δ* can be provided as 0.61 and 0.69, respectively [36].

## 3. The Proposed FMEA Model for Railway Train Risk Prioritization

In this section, a comprehensive FMEA model based on cumulative prospect theory and type-2 intuitionistic fuzzy VIKOR is developed to analyze the risk of railway train and rank the crucial components. 

**Figure 1:**
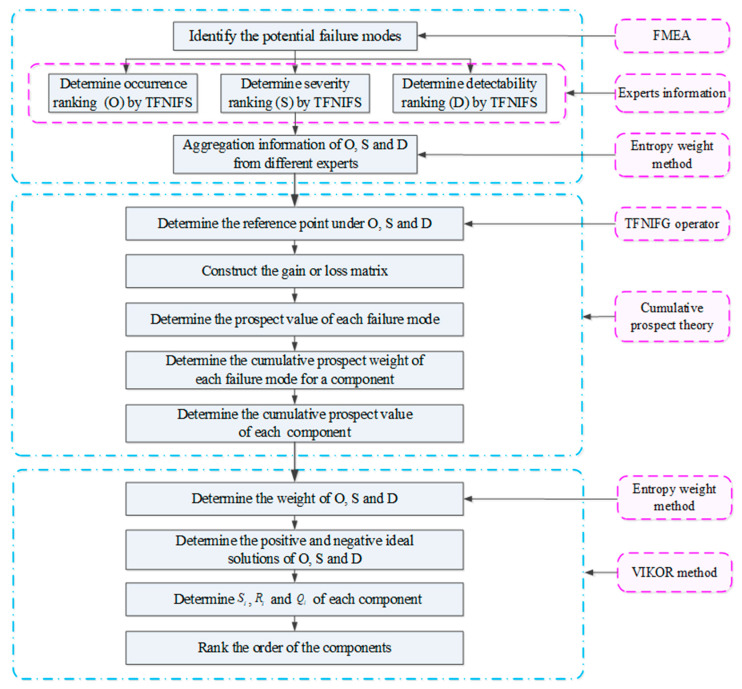
Flowchart of the proposed FMEA model.

The risk prioritization in FMEA model can be treated as a MCDM problem, and the risk factor O, S and D can be regarded as a group of decision criteria. As a result, the procedure of resolving this FMEA problem can be considered as a process of risk prioritization where uncertainty and FMEA team members’ preference and psychological behavior are both taken into account. The general steps for this proposed FMEA model can be illustrated as Figure 1 and the systematic procedures can be briefly introduced as follows. 

### 3.1. Determine and Aggregate the FMEA Decision Value by Type-2 Intuitionistic Fuzzy Number

In the process of failure mode and component risk prioritization, we can assume that there are *m* alternatives, namely system components denoted by *A_i_* (*i* = 1, 2, …, *m*), *t* failure modes for a component *A_i_*, presented by *FM_is_* (*is* = 1, 2, …, *t*), 3 criteria, namely risk factor O, S and D, indicated by *C_j_* (*j* = 1, 2, 3), and *l* decision makers, namely FMEA team members, expressed by *DM_k_* (*k* = 1, 2, …, *l*). Let $r_{isj}^{k}$ be the decision value for the failure mode *FM_is_* on criterion *C_j_* about the FMEA team member *DM_k_*. Moreover, in light of uncertainty of the risk factor O, S and D, the decision value can be expressed by TFNIFNs presented as $r_{isj}^{k}=\left(\left(al_{isj}^{k},am_{isj}^{k},ar_{isj}^{k}\right),\left(bl_{isj}^{k},bm_{isj}^{k},br_{isj}^{k}\right)\right)$.

Every FMEA team member is required to give the risk score of the failure mode *FM_is_* on criterion *C_j_* by TFNIFNs, as provided in Table 1, Table 2 and Table 3, respectively.

With respect to the different FMEA team members, the different risk score information needs to be aggregated based on the team members’ weight. In the literature [43], there are various approaches to calculate the importance weight of different expert, such as AHP method [44], distance-based method [45], SWARA method [46] and entropy weight method [23,30], etc. The entropy weight method has been widely used in the MCDM problems, and it can illustrate relative information more objectively [23,30]. Therefore, the entropy weight method is applied to determine the weights of FMEA team members in this paper. The process to determine the FMEA team members’ weights can be briefly explained as follows.

Step 1: Determine the TFNIFG operator ${\tilde{r}}_{j}^{k}$ of each FMEA team member under different risk factors.

(20) $$\begin{array}{l} {\tilde{r}}_{j}^{k}=(\left(\prod_{is=1}^{N}{\left(al_{isj}^{k}\right)}^{1/N},\prod_{is=1}^{N}{\left(am_{isj}^{k}\right)}^{1/N},\prod_{is=1}^{N}{\left(ar_{isj}^{k}\right)}^{1/N}\right),\\ \left(\left(1-\prod_{is=1}^{N}{\left(1-bl_{isj}^{k}\right)}^{1/N}\right),\left(1-\prod_{is=1}^{N}{\left(1-bm_{isj}^{k}\right)}^{1/N}\right),\left(1-\prod_{is=1}^{N}{\left(1-br_{isj}^{k}\right)}^{1/N}\right)\right)) \end{array}$$

Step 2: Determine the risk score information entropy of each FMEA team member under different risk factors.

(21) $$e_{j}^{k}=-\frac{1}{\ln\left(N\right)}\sum_{is=1}^{N}\frac{d\left(r_{isj}^{k},{\tilde{r}}_{j}^{k}\right)}{\sum_{is=1}^{N}d\left(r_{isj}^{k},{\tilde{r}}_{j}^{k}\right)}\ln\left(\frac{d\left(r_{isj}^{k},{\tilde{r}}_{j}^{k}\right)}{\sum_{is=1}^{N}d\left(r_{isj}^{k},{\tilde{r}}_{j}^{k}\right)}\right)$$

Step 3: Determine the importance weight of different FMEA team member under different risk factors.

(22) $$w_{j}^{k}=\frac{1-e_{j}^{k}}{\sum_{k=1}^{l}1-e_{j}^{k}}$$

Consequently, the aggregated risk score information of failure mode *FM_is_* can be gathered as $r_{isj}=\left(\left(al_{isj},am_{isj},ar_{isj}\right),\left(bl_{isj},bm_{isj},br_{isj}\right)\right)$.

### 3.2. Determine the Cumulative Prospect Value of Each Component

In this part, different failure modes of a component can be regarded as the different performance states of the component. As for the component risk, it refers to the different performance states of a component with different probability. As a result, the component risk associated with different failure modes can be determined based on the cumulative prospect theory. The procedure to calculate the cumulative prospect value of each component can be summarized as follows.

Step 1: Determine the reference points under different risk factors.

With respect to the risk score information of different failure mode, it expresses as TFNIFN. Consequently, the reference points can be designed as the TFNIFG operator for a set of risk score information. Let *r_j_* = ((*al_j_*, *am_j_*, *ar_j_*), (*bl_j_*, *bm_j_*, *br_j_*)) be the reference point under risk factor *C_j_*. *r_j_* can be expressed as

(23) $$\begin{array}{l} {\tilde{r}}_{j}^{}=(\left(\prod_{is=1}^{N}{\left(al_{isj}^{}\right)}^{1/N},\prod_{is=1}^{N}{\left(am_{isj}^{}\right)}^{1/N},\prod_{is=1}^{N}{\left(ar_{isj}^{}\right)}^{1/N}\right),\\ \left(\left(1-\prod_{is=1}^{N}{\left(1-bl_{isj}^{}\right)}^{1/N}\right),\left(1-\prod_{is=1}^{N}{\left(1-bm_{isj}^{}\right)}^{1/N}\right),\left(1-\prod_{is=1}^{N}{\left(1-br_{isj}^{}\right)}^{1/N}\right)\right)) \end{array}$$

Step 2: Determine and construct the gain or loss matrix.

After determining the reference point, the gain or loss value of a failure mode can be constructed based on Hamming distance between aggregated risk score information and reference point.

(24) $$v\left(\Delta r_{isj}\right)=\left\{ \begin{array}{l} d{\left(r_{isj}^{},r_{j}\right)}^{0.88},r_{isj}^{}\geq r_{j}\\ -2.25d{\left(r_{isj}^{},r_{j}\right)}^{0.88},r_{isj}^{}<r_{j} \end{array}\right.$$

Consequently, the gain or loss matrix of the failure modes can be expressed as follows.

(25) $$\begin{array}{c} \;\begin{array}{cccc} C_{1} & \;C_{2} & \;\cdots & \;C_{n} \end{array}\\ V=\begin{array}{c} A_{1}\;FM_{11}\\ A_{1}\;FM_{12}\\ \vdots \\ A_{m}\;FM_{mt} \end{array}\left[\begin{array}{cccc} v\left(\Delta r_{111}\right) & v\left(\Delta r_{112}\right) & \cdots & v\left(\Delta r_{11n}\right)\\ v\left(\Delta r_{121}\right) & v\left(\Delta r_{122}\right) & \cdots & v\left(\Delta r_{12n}\right)\\ \vdots & \vdots & \ddots & \vdots \\ v\left(\Delta r_{mt1}\right) & v\left(\Delta r_{mt2}\right) & \cdots & v\left(\Delta r_{mtn}\right) \end{array}\right] \end{array}$$

Step 3: Determine the cumulative prospect weight of each failure mode for a component.

Based on the step 2, the importance weight of each failure mode for a component can be obtained based on the cumulative prospect theory. The cumulative prospect weight is the promotion of the traditional prospect weight as it considers the cumulative probability rather than a single probability. Therefore, the order of gain or loss value of failure modes for a component can be determined by increasing ranking. Let −*m* ≤ *is* ≤ *n*, the ranking order can be denoted as $v\left(\Delta r_{-m}\right)\leq v\left(\Delta r_{-m+1}\right)\leq \ldots \leq v\left(\Delta r_{isj}\right)\leq \ldots \leq v\left(\Delta r_{n-1}\right)\leq v\left(\Delta r_{n}\right)$. The failure mode *FM_is_* and probability *p_is_* under the risk factor *C_j_* is associated with $v\left(\Delta r_{isj}\right)$. Thus, the cumulative prospect weight of each failure mode can be determined as follows.

(26) $$\begin{array}{l} \Pi_{is}^{+}=w^{+}\left(p_{is}+p_{is+1}+\cdots +p_{n}\right)-w^{+}\left(p_{is+1}+p_{is+2}+\cdots +p_{n}\right),0\leq is<n\\ \Pi_{is}^{-}=w^{-}\left(p_{-m}+p_{-m+1}+\cdots +p_{is}\right)-w^{-}\left(p_{-m}+p_{-m+1}+\cdots +p_{is-1}\right),-m<is\leq 0 \end{array}$$

In which the weight function *w*^+^ and *w*^−^ can be determined in accordance with Equations (18) and (19), $\Pi_{n}^{+}=w^{+}\left(p_{n}\right)$ and $\Pi_{m}^{-}=w^{-}\left(p_{m}\right)$.

Step 4: Determine the cumulative prospect value of each component.

Based on the gain or loss matrix and cumulative prospect weight of the failure modes, the cumulative prospect value of a component can be expressed as follows.

(27) $$V_{A_{ij}}=\sum_{is=-m}^{0}v\left(\Delta r_{isj}\right)\Pi_{is}^{-}+\sum_{is=1}^{n}v\left(\Delta r_{isj}\right)\Pi_{is}^{+}$$

### 3.3. Determine the Component Risk Prioritization by VIKOR

After determining the cumulative prospect value of each component under different risk factors, the comprehensive risk ranking and prioritization of the system component can be determined based on the VIKOR method. The importance weight of different risk factors can be calculated by entropy weight method [23]. The procedure to determine the comprehensive risk prioritization of each component can be summarized as follows.

Step 1: Determine the importance weight of risk factor O, S and D.

Based on the cumulative prospect value of components, the risk information entropy of different factors can be expressed as follows.

(28) $$e_{j}=-\frac{1}{\ln\left(m\right)}\sum_{i=1}^{m}\frac{d\left(V_{A_{ij}},V_{A_{j}}\right)}{\sum_{i=1}^{m}d\left(V_{A_{ij}},V_{A_{j}}\right)}\ln\left(\frac{d\left(V_{A_{ij}},V_{A_{j}}\right)}{\sum_{i=1}^{m}d\left(V_{A_{ij}},V_{A_{j}}\right)}\right)$$

In which $V_{A_{ij}}$ is the cumulative prospect mean value of each component and can be calculated as follows.

(29) $$V_{A_{j}}=\frac{1}{m}\sum_{i=1}^{m}V_{A_{ij}}$$

Hence, the importance weight of risk factor O, S and D can be determined as

(30) $$w_{j}=\frac{1-e_{j}}{\sum_{j=1}^{n}1-e_{j}}$$

Step 2: Determine the positive ideal solution and negative ideal solution.

Based on the Equation (9), the positive ideal solution $V_{A_{j}}^{+}$ and negative ideal solution $V_{A_{j}}^{-}$ under risk factor O, S and D can be obtained.

Step 3: Determine the maximizing group utility, minimizing individual regret and compromise solution.

Based on the Equation (10) to Equation (13), the maximizing group utility *S_i_*, minimizing individual regret *R_i_* and compromise solution *Q_i_* can be obtained.

Step 4: Determine the risk ranking and prioritization of each component.

The risk ranking and prioritization order of each component can be obtained based on the ascending order by the compromise solution *Q_i_*.

## 4. An Illustrative Example

In this section, an illustrative risk analysis case study of a specific railway train bogie system has been applied to demonstrate the application and feasibility of the proposed FMEA model. Further-more, a comparison study is also conducted to validate the effectiveness of the new FMEA model.

### 4.1. Calculation of the Risk Prioritization for Railway Train Bogie System

Step 1: Determine and aggregate the FMEA decision value.

Bogie system is one of the most major complex mechatronic part of railway train and can be easily prone to fail. Bogie system can account for a substantial 21.1% based on accumulation of failure data in a couple years [47]. In this paper, a specific railway train bogie system is applied to cope with the proposed FMEA model. Twenty-eight components and seventy-three failure modes are selected for the calculation, which can be *A_i_* (*i* = 1, 2, …, 28) and *FM_is_* (*is* = 1, 2, …, *t*, i*is = 73). The components and failure modes can be provided in Table 4 and Table 5, respectively. A FMEA team consists of three members: design manufacturer, train crew and maintenance personnel. They are invited to give the risk score of the failure mode under risk factor O, S and D, and can be expressed by *DM_k_* (*k* = 1, 2, 3). 

Based on the Table 1, Table 2 and Table 3, every FMEA team member gives the risk score of the failure mode *FM_is_* under the risk factor O, S and D. Table 6 provides the risk score of partial failure modes. Then, importance weights of the FMEA team member can be calculated as 0.2822, 0.3268 and 0.3910 under risk factor O, 0.2455, 0.2406 and 0.5139 under risk factor S and 0.3678, 0.3538 and 0.2784 under risk factor D, respectively. As a result, the aggregated risk score of partial failure modes can be obtained according to Equations (20)–(22) in Section 3.1. Table 7 provides the aggregated risk score of partial failure modes.

Step 2: Determine the cumulative prospect value of each component.

On account of the aggregated risk score of all the failure modes, the reference points under risk factor O, S and D can be determined as ((0.21,0.25,0.37), (0.74,1.0,1.0)), ((0.45,0.55,0.65), (0.45,0.55,0.65)) and ((0.33,0.42,0.52), (0.58,1.0,1.0)), respectively. Then, the gain or loss matrix of failure modes can be obtained based on the Hamming distance between the aggregated risk scores and reference points. Table 8 provides the gain or loss matrix of partial failure modes.

According to the increasing ranking order of gain or loss value of the failure modes for a component, the cumulative prospect weight of each failure mode can be determined in accordance with Equations (26) and (27) in Section 3.2. The cumulative prospect weight of partial failure modes can be shown in Table 9. Thus, the cumulative prospect value of each component can be obtained based on the gain or loss matrix and cumulative prospect weight of failure modes. Table 10 provides the cumulative prospect value of partial bogie system components.

Step 3: Determine the component risk prioritization.

**Figure 2:**
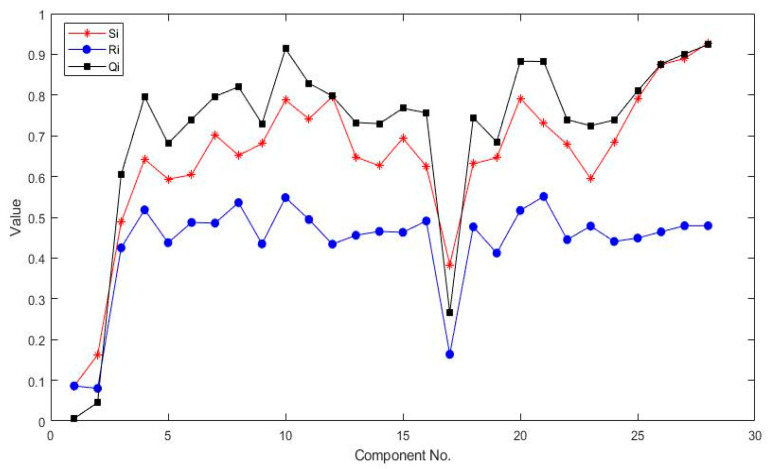
*S_i_*, *R_i_* and *Q_i_* value of bogie system components.

Based on the Equation (28) to Equation (30), we can obtain the weight under risk factor O, S and D as 0.25, 0.18 and 0.57. Then, the maximizing group utility *S_i_*, minimizing individual regret *R_i_* and compromise solution *Q_i_* can be determined by the VIKOR approach, which can be shown in Figure 2. Table 11 provides the *S_i_*, *R_i_* and *Q_i_* value of partial bogie system components. Therefore, the risk ranking and prioritization order can be obtained by the increasing order of *Q_i_* value.

On the basis of the increasing order of the compromise solution *Q_i_* of bogie system components, the risk prioritization ranking result of the top ten components can be Frame assembly > Axle > Drawbar > Wheel > Bearing > Central axis > Parking brake unit > Air spring > Gearbox assembly > Anti-rolling torsion bar. Consequently, the component frame assembly has the top risk prioritization, because frame assembly is the main body of railway train bogie system. Any failures will cause the derailment of the train and results in irretrievable damage. Thus, reliability design of the frame assembly has been always attached great importance.

Furthermore, the crack of axle and drawbar can also lead to the derailment of the train. The microcrack of axle and drawbar can cause the decline of the railway train operation stability and endanger the safety of passengers. The wear of the drawbar counts a large proportion of the fault record and sometimes affect the railway train operation. As a consequence, the component axle and drawbar has the second and third risk prioritization.

In addition, the parking brake unit is an important component to prevent sliding at the parking time when passengers are getting on and off the train. Once the parking brake fails, it can bring about train sliding, and subsequently, passenger injuries. Therefore, FMEA team members give it a high-risk score, and the parking brake has the seventh risk prioritization.

### 4.2. Comparisons and Discussion

**Figure 3:**
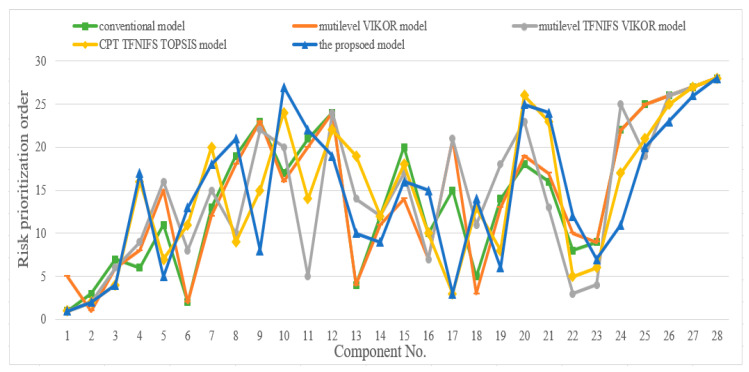
Risk prioritization order of failure modes of bogie system components.

To verify the efficiency of the extended FMEA model, a comparison study is performed with other approaches based on the example mentioned above. The extended FMEA model based on cumulative prospect theory and type-2 intuitionistic fuzzy VIKOR approach has been proposed to improve the accuracy of conventional FMEA by considering the uncertainty, experts’ risk sensitiveness and decision-making psychological behavior. As a result, the conventional FMEA model (conventional model), the multilevel FMEA based on VIKOR model (multilevel VIKOR model), multilevel FMEA with TFNIFS VIKOR model (multilevel TFNIFS VIKOR model) and FMEA with cumulative prospect theory, TFNIFS and TOPSIS model (CPT TFNIFS TOPSIS model) are selected for comparison study to find out the advantages of the proposed FMEA model. The risk prioritization outcomes of the illustrative models are represented in Figure 3.

As shown in Figure 3, the overall prioritization trend of the proposed model essentially agrees with the illustrative models. There are some differences among these models, and the explanations can be given as follows.

The first comparison study is conducted with the outcome derived based on the conventional FMEA model. There are significant differences between the risk prioritization outcomes by the two FMEA models. For instance, the component primary spring has the second risk prioritization in the conventional model, however, it ranks thirteenth when the proposed model is used. The failure of the primary spring practically affects the passenger comfort instead of operation safety. Moreover, the component central axis and drawbar has the fourteenth and fifteenth risk prioritization in the conventional model, which is inconsistent with the actual situation. Central axis and drawbar are the crucial components associated with the safety operation. Based on the proposed model, central axis and drawbar ranks sixth and third, respectively. Therefore, the outcome derived from the conventional model can be inaccurate to some extent. The inaccurate outcomes can be explained as follows:

- The uncertainty, experts’ risk sensitiveness and decision-making psychological behavior are not considered in the conventional model.
- The multiplication of risk factor O, S and D can be questionable.

The second comparative analysis is performed with the outcome obtained by the multilevel VIKOR model. There are also numerous distinctions between the two models. For example, the major difference is that the component frame assembly ranks fifth in the multilevel VIKOR model. However, it has the top risk prioritization when the proposed method is adopted, which is consistent with the other three models. In addition, the component primary spring still has the second risk prioritization in the multilevel VIKOR model and is inconsistent with the actual situation. Furthermore, the component axle box body and traction motor have the eighth and seventh risk prioritization in the multilevel VIKOR model, respectively. The failure of axle box body merely affects the passenger comfort and the failure of traction motor will decline the operation stability. According to the proposed model, the axle box body and traction motor rank seventeenth and fifteenth, respectively, which is consistent with the actual situation. The component brake pad has a significant relationship with the operation safety. It ranks eleventh in the proposed model while it barely ranks twenty-second in the multilevel VIKOR model. As a result, the distinction between the two models is that the multilevel VIKOR model considers the VIKOR for multiplication of risk factors but does not think about the uncertainty, experts’ risk sensitiveness and decision-making psychological behavior.

The third comparative analysis is implemented with multilevel TFNIFS VIKOR model. The major difference between the two models is that the component central axis and drawbar has the eighteenth and twenty-first risk prioritization in the multilevel TFNIFS VIKOR model, respectively, and is inconsistent with the actual situation. Moreover, the component air spring has the twenty-second risk prioritization in the multilevel TFNIFS VIKOR model while it ranks eighth in the proposed model. The air spring is the significant component related to operation safety, that needs much attention to keep its reliability. Consequently, the difference between the two models indicates the importance of considering the experts’ risk sensitiveness and decision-making psychological behavior in risk analysis. The last comparative analysis is carried out with CPT TFNIFS TOPSIS model. From Figure 3, the two models share the same risk prioritization about the top four components. However, the risk prioritization of the component air spring and anti-rolling torsion bar in the CPT TFNIFS TOPSIS model vary significantly with the outcomes in the proposed model. As important as air spring, the anti-rolling torsion bar ensure the operation safety when train is travelling and negotiating curve. Air spring and anti-rolling torsion bar ranks eighth and tenth when proposed method is adopted. However, air spring and anti-rolling torsion bar ranks fifteenth and nineteenth in the CPT TFNIFS TOPSIS model, respectively. Hence, the similarities between the two models are that they both consider the same FMEA framework with the MCDM method, uncertainty, experts’ risk sensitiveness and decision-making psychological behavior. Nevertheless, the difference between them is that the VIKOR approach is adopted as the MCDM method in the proposed model as opposed to TOPSIS for the other. Fei et al and Hsu [30,31] illustrates the superiority and flexibility of the VIKOR approach compared with TOPSIS.

Therefore, the comparison studies mentioned above confirm the validity of the proposed FMEA model. The improvement of the enhanced FMEA model based on type-2 intuitionistic fuzzy numbers, VIKOR and cumulative prospect theory can well cope with uncertainty, experts’ risk sensitiveness, decision-making psychological behavior and risk factor fusion problem for the railway train risk prioritization.

## 5. Conclusions

In this paper, an extended FMEA model is proposed for the railway train risk prioritization based on cumulative prospect theory and type-2 intuitionistic fuzzy VIKOR approach. Firstly, the FMEA framework is applied to examine the vital and risky failure modes and components for the railway train operation. Then, the three risk factors O, S and D are evaluated based on the triangle fuzzy number intuitionistic fuzzy numbers to handle the uncertainty in the risk evaluation for each failure mode. Next, cumulative prospect theory is utilized because it can take into account the FMEA team members’ risk sensitiveness and psychological behavior in the analysis process. Then, TFNIFG operator is applied as the reference point to determine the cumulative prospect value of each failure mode. In addition, the VIKOR method is used for information fusion of the risk factor O, S and D. Furthermore, the entropy weighting method is also adopted to determine FMEA team members’ importance weights and risk factor weights. Finally, the risk prioritization of each component can be obtained. An illustrative example of railway train bogie system is performed to demonstrate the effectiveness of the proposed FMEA model.

Based on the calculation outcome, a comparison study is also carried out with other approaches to validate the effectiveness of the enhanced FMEA model. In accordance with the comparison study, it found that the proposed FMEA model can provide more reasonable and precise results for the railway train risk prioritization.

In regard to future research directions, it is recommended that the multiplication problems for the different risk factors should be handled not only by the MCDM method, but also by evidence theory. In addition, the dynamic FMEA model can be integrated into this problem by considering the full life-cycle activities of the railway train. Furthermore, we will extend the cumulative prospect theory with a number of reference points by taking into account different dimensional risk information.

## Figures and Tables

**Table 1:** Linguistic variables for the risk factor occurrence (O).

Linguistic Variables	Rank	TFNIFNs
Very low	1, 2	((0.1,0.1,0.2), (0.9,1.0,1.0))
Low	3, 4	((0.2,0.3,0.4), (0.7,0.8,0.9))
Medium	5, 6	((0.4,0.5,0.6), (0.5,0.6,0.7))
High	7, 8	((0.6,0.7,0.8), (0.3,0.4,0.5))
Very high	9, 10	((0.8,0.9,1.0), (0.1,0.2,0.3))

**Table 2:** Linguistic variables for the risk factor severity (S).

Linguistic Variables	Rank	TFNIFNs
Very minor	1, 2	((0.1,0.1,0.2), (0.9,1.0,1.0))
Minor	3, 4	((0.2,0.3,0.4), (0.7,0.8,0.9))
Medium	5, 6	((0.4,0.5,0.6), (0.5,0.6,0.7))
Major	7, 8	((0.6,0.7,0.8), (0.3,0.4,0.5))
Hazardous	9, 10	((0.8,0.9,1.0), (0.1,0.2,0.3))

**Table 3:** Linguistic variables for the risk factor detection (D).

Linguistic Variables	Rank	TFNIFNs
Very easy	1, 2	((0.1,0.1,0.2), (0.9,1.0,1.0))
Easy	3, 4	((0.2,0.3,0.4), (0.7,0.8,0.9))
Medium	5, 6	((0.4,0.5,0.6), (0.5,0.6,0.7))
Hard	7, 8	((0.6,0.7,0.8), (0.3,0.4,0.5))
Very hard	9, 10	((0.8,0.9,1.0), (0.1,0.2,0.3))

**Table 4:** Bogie system components selected for the calculation.

No.	Component	No.	Component
1	Frame assembly	15	Coupling
2	Axle	16	Traction motor
3	Wheel	17	Drawbar
4	Axle box body	18	Traction frame
5	Bearing	19	Central axis
6	Primary spring	20	Central pin
7	Vertical shock absorber	21	Lateral buffer device
8	Lateral stop	22	Tread Braking Unit
9	Air spring	23	Parking brake unit
10	Height adjustment device	24	Brake pad
11	Differential pressure valve	25	Flange lubrication device
12	Lateral shock absorber	26	Grounding device
13	Anti-rolling torsion bar	27	RFID
14	Gearbox assembly	28	Temperature sensor

**Table 5:** Bogie system components selected for the calculation.

No.	Component No.	Failure Mode	No.	Component No.	Failure Mode
1	1	Microcrack	38	14	Oil leak
2	1	Crack	39	14	Low oil level
3	2	Microcrack	40	14	Microcrack
4	2	Crack	41	14	Crack
5	3	Crack	42	14	Abnormal sound
6	3	Tread scratch	43	14	Gear damage
7	3	Tread peeling	44	14	Gear crack
8	3	Tread crack	45	15	Microcrack
9	3	Wheel wear	46	15	Crack
10	4	Microcrack	47	15	Loosening
11	4	Wear	48	16	Abnormal vibration
12	4	Crack	49	16	Over-temperature
13	4	Abnormal temperature	50	16	Bearing crack
14	5	Scratch	51	16	Internal wiring damage
15	5	Microcrack	52	16	Stop turning
16	5	Crack	53	17	Crack
17	5	Abnormal temperature	54	17	Microcrack
18	6	Wear	55	17	Wear
19	6	Microcrack	56	18	Crack
20	6	Crack	57	18	Microcrack
21	7	Loosening	58	19	Crack
22	7	Oil leak	59	19	Microcrack
23	8	Microcrack	60	20	Wear
24	8	Crushed	61	20	Microcrack
25	9	Scratch	62	20	Crack
26	9	Crack	63	21	Crack
27	9	Air leak	64	21	Microcrack
28	10	Block	65	22	Brake failure
29	10	Loosening	66	23	Brake failure
30	11	Block	67	24	Wear
31	12	Oil leak	68	24	Unable to brake
32	12	Ageing	69	25	No oil supply
33	13	Loosening	70	25	Stent crack
34	13	Deformation	71	26	Open circuit
35	13	Microcrack	72	27	Unable to transmit
36	13	Crack	73	28	Unable to detect
37	14	Abnormal oil			

**Table 6:** Bogie system components selected for the calculation.

No.	Component No.	*p*	DM1 for Factor O	DM1 for Factor S	DM1 for Factor D	DM2 for Factor O	DM2 for Factor S	DM2 for Factor D	DM3 for Factor O	DM3 for Factor S	DM3 for Factor D
3	2	0.9	((0.6,0.7,0.8), (0.3,0.4,0.5))	((0.6,0.7,0.8), (0.3,0.4,0.5))	((0.6,0.7,0.8), (0.3,0.4,0.5))	((0.4,0.5,0.6), (0.5,0.6,0.7))	((0.6,0.7,0.8), (0.3,0.4,0.5))	((0.6,0.7,0.8), (0.3,0.4,0.5))	((0.4,0.5,0.6), (0.5,0.6,0.7))	((0.6,0.7,0.8), (0.3,0.4,0.5))	((0.4,0.5,0.6), (0.5,0.6,0.7))
4	2	0.1	((0.1,0.1,0.2), (0.9,1.0,1.0))	((0.8,0.9,1.0), (0.1,0.2,0.3))	((0.6,0.7,0.8), (0.3,0.4,0.5))	((0.1,0.1,0.2), (0.9,1.0,1.0))	((0.8,0.9,1.0), (0.1,0.2,0.3))	((0.6,0.7,0.8), (0.3,0.4,0.5))	((0.1,0.1,0.2), (0.9,1.0,1.0))	((0.8,0.9,1.0), (0.1,0.2,0.3))	((0.6,0.7,0.8), (0.3,0.4,0.5))
5	3	0.05	((0.6,0.7,0.8), (0.3,0.4,0.5))	((0.6,0.7,0.8), (0.3,0.4,0.5))	((0.6,0.7,0.8), (0.3,0.4,0.5))	((0.6,0.7,0.8), (0.3,0.4,0.5))	((0.4,0.5,0.6), (0.5,0.6,0.7))	((0.4,0.5,0.6), (0.5,0.6,0.7))	((0.4,0.5,0.6), (0.5,0.6,0.7))	((0.4,0.5,0.6), (0.5,0.6,0.7))	((0.4,0.5,0.6), (0.5,0.6,0.7))
6	3	0.05	((0.4,0.5,0.6), (0.5,0.6,0.7))	((0.8,0.9,1.0), (0.1,0.2,0.3))	((0.6,0.7,0.8), (0.3,0.4,0.5))	((0.2,0.3,0.4), (0.7,0.8,0.9))	((0.8,0.9,1.0), (0.1,0.2,0.3))	((0.4,0.5,0.6), (0.5,0.6,0.7))	((0.2,0.3,0.4), (0.7,0.8,0.9))	((0.8,0.9,1.0), (0.1,0.2,0.3))	((0.4,0.5,0.6), (0.5,0.6,0.7))
7	3	0.05	((0.2,0.3,0.4), (0.7,0.8,0.9))	((0.6,0.7,0.8), (0.3,0.4,0.5))	((0.6,0.7,0.8), (0.3,0.4,0.5))	((0.2,0.3,0.4), (0.7,0.8,0.9))	((0.6,0.7,0.8), (0.3,0.4,0.5))	((0.6,0.7,0.8), (0.3,0.4,0.5))	((0.1,0.1,0.2), (0.9,1.0,1.0))	((0.4,0.5,0.6), (0.5,0.6,0.7))	((0.4,0.5,0.6), (0.5,0.6,0.7))
8	3	0.84	((0.8,0.9,1.0), (0.1,0.2,0.3))	((0.4,0.5,0.6), (0.5,0.6,0.7))	((0.4,0.5,0.6), (0.5,0.6,0.7))	((0.8,0.9,1.0), (0.1,0.2,0.3))	((0.4,0.5,0.6), (0.5,0.6,0.7))	((0.4,0.5,0.6), (0.5,0.6,0.7))	((0.8,0.9,1.0), (0.1,0.2,0.3))	((0.4,0.5,0.6), (0.5,0.6,0.7))	((0.4,0.5,0.6), (0.5,0.6,0.7))
9	3	0.01	((0.1,0.1,0.2), (0.9,1.0,1.0))	((0.8,0.9,1.0), (0.1,0.2,0.3))	((0.6,0.7,0.8), (0.3,0.4,0.5))	((0.1,0.1,0.2), (0.9,1.0,1.0))	((0.8,0.9,1.0), (0.1,0.2,0.3))	((0.8,0.9,1.0), (0.1,0.2,0.3))	((0.1,0.1,0.2), (0.9,1.0,1.0))	((0.8,0.9,1.0), (0.1,0.2,0.3))	((0.8,0.9,1.0), (0.1,0.2,0.3))

**Table 7:** Aggregated risk score of partial failure modes.

No.	Component No.	*p*	O	S	D
3	2	0.9	((0.45,0.55,0.65), (0.44,0.54,0.64))	((0.6,0.7,0.8), (0.3,0.4,0.5))	((0.54,0.64,0.74), (0.35,0.45,0.55))
4	2	0.1	((0.1,0.1,0.2), (0.9,1.0,1.0))	((0.8,0.9,1.0), (0.1,0.2,0.3))	((0.6,0.7,0.8), (0.3,0.4,0.5))
5	3	0.05	((0.52,0.62,0.72), (0.37,0.47,0.57))	((0.45,0.55,0.65), (0.44,0.54,0.64))	((0.47,0.57,0.67), (0.42,0.52,0.62))
6	3	0.05	((0.26,0.36,0.46), (0.64,0.74,0.84))	((0.8,0.9,1.0), (0.1,0.2,0.3))	((0.47,0.57,0.67), (0.42,0.52,0.62))
7	3	0.05	((0.16,0.22,0.32), (0.77,0.87,0.93))	((0.49,0.59,0.69), (0.40,0.50,0.60))	((0.54,0.64,0.74), (0.35,0.45,0.55))
8	3	0.84	((0.8,0.9,1.0), (0.1,0.2,0.3))	((0.4,0.5,0.6), (0.5,0.6,0.7))	((0.4,0.5,0.6), (0.5,0.6,0.7))
9	3	0.01	((0.1,0.1,0.2), (0.9,1.0,1.0))	((0.8,0.9,1.0), (0.1,0.2,0.3))	(0.72,0.82,0.92), (0.17,0.27,0.37)

**Table 8:** Gain or loss matrix of partial failure modes.

No.	Component No.	*p*	O	S	D
3	2	0.9	0.38	0.19	0.37
4	2	0.1	−0.28	0.41	0.44
5	3	0.05	0.45	−0.03	−0.69
6	3	0.05	0.17	0.40	−0.69
7	3	0.05	−0.20	0.08	0.38
8	3	0.84	0.71	−0.14	−0.51
9	3	0.01	−0.28	0.40	0.56

**Table 9:** Cumulative prospect weight of partial failure modes.

No.	Component No.	*p*	O	S	D
3	2	0.9	0.71	0.81	0.81
4	2	0.1	0.17	0.19	0.19
5	3	0.05	0.05	0.06	0.11
6	3	0.05	0.07	0.09	0.06
7	3	0.05	0.09	0.05	0.09
8	3	0.84	0.64	0.71	0.66
9	3	0.01	0.04	0.05	0.06

**Table 10:** Cumulative prospect weight of partial components.

Component No.	O	S	D
1	0.468144	−0.04211	−0.39385
2	0.298719	−0.09364	−0.60525
3	0.281249	−0.22827	−0.42217
4	0.336425	−0.1147	−0.5351
5	0.030094	−0.15011	−0.5314
6	0.052312	−0.0584	−0.46359
7	0.098971	−0.40377	−0.41591
8	0.468144	−0.04211	−0.39385
9	0.298719	−0.09364	−0.60525

**Table 11:** *S_i_*, *R_i_* and *Q_i_* value of partial components.

Component No.	O	S	D
1	0.468144	−0.04211	−0.39385
2	0.298719	−0.09364	−0.60525
3	0.281249	−0.22827	−0.42217
4	0.336425	−0.1147	−0.5351
5	0.030094	−0.15011	−0.5314
6	0.052312	−0.0584	−0.46359
7	0.098971	−0.40377	−0.41591
8	0.468144	−0.04211	−0.39385
9	0.298719	−0.09364	−0.60525
